# Effects of Dietary Ammonium Chloride Supplementation on the Lipidome and Volatile Flavor Compounds in the Subcutaneous Adipose Tissue of Tibetan Sheep

**DOI:** 10.3390/foods15030554

**Published:** 2026-02-04

**Authors:** Juyuan He, Anum Ali Ahmad, Jiancui Wang, Qingling Ma, Shengzhen Hou, Zenghai Luo, Chao Yang

**Affiliations:** 1 State Key Laboratory of Plateau Ecology and Agriculture, Qinghai University, Xining 810016, China; hjy3046@163.com (J.H.); qhdxhsz@163.com (S.H.); 2 The Roslin Institute, The University of Edinburgh, Easter Bush Campus, Midlothian, Edinburgh EH25 9RG, UK; aahmad3@ed.ac.uk; 3 College of Agriculture and Animal Husbandry, Qinghai University, Xining 810016, China; jiancuiwang@163.com (J.W.); qinglingma@126.com (Q.M.); 4 Department of Animal Science, Qinghai Agri-Animal Husbandry Vocational College, Xining 812100, China

**Keywords:** ammonium chloride, subcutaneous adipose tissue, tissue morphology, fatty acids, lipidomics, volatile flavor compounds

## Abstract

As a source of non-protein nitrogen, ammonium chloride (NH_4_Cl) is widely utilized in ruminant diets to reduce feed costs. However, the impact of its supplementation level on the flavor of sheep meat remains unclear, despite the known influence of fat on meat flavor. This study aimed to investigate the effects of dietary NH_4_Cl supplementation levels on the lipidome and flavor compounds of subcutaneous adipose tissue in Tibetan sheep, providing a scientific basis for dietary optimization in Tibetan sheep farming. Eighty 2-month-old early-weaned Tibetan lambs were selected and randomly allocated into four groups, fed diets supplemented with 0% (N0 group), 1.49% (N1 group), 2.24% (N2 group), and 3.01% (N3 group) NH_4_Cl for an experimental period of 105 days. The study conducted histomorphological observations, lipidomics analysis, and determination of flavor compounds. The results showed that NH_4_Cl supplementation significantly reduced (*p* < 0.05) the contents of various unsaturated fatty acids and n-3 polyunsaturated fatty acids (n-3 PUFA) in the subcutaneous adipose tissue of Tibetan sheep. Specifically, the total saturated fatty acid (total SFA) content in the N3 group was significantly higher than that in the other groups, while the total monounsaturated fatty acid (total MUFA) content was significantly lower than that in the N1 and N2 groups (*p* < 0.05). The absolute contents of phosphatidylcholine (PC), phosphatidylethanolamine (PE), and the sum of triglycerides (TGs) and diglycerides (DGs) in the N3 group were significantly higher (*p* < 0.05) than those in the other groups. Regarding flavor compounds, the contents of ketone aroma compounds, such as 2-propanone and 2-butanone monomer, were significantly higher (*p* < 0.05) in the N0 group than in the other groups. The ammonia content in the N1 and N3 groups was significantly higher (*p* < 0.05) than that in the N0 and N2 groups, while the allyl sulfide content in the N2 group was significantly higher (*p* < 0.05) than that in the other groups. Correlation analysis revealed that the majority of TG and DG differential lipids were significantly positively correlated with allyl sulfide, and most differential lipids belonging to the PC, PE, and hexosylceramide (Hex1Cer) classes were significantly positively correlated with ammonia (|r| ≥ 0.80, *p* < 0.01). Conversely, PC (16:0_18:3) exhibited significant negative correlations with multiple beneficial aroma compounds (|r| ≥ 0.80, *p* < 0.01). The study indicates that dietary NH_4_Cl supplementation levels exceeding 2.24% are associated with alterations in lipid metabolism and reduced synthesis of unsaturated fatty acids and beneficial flavor compounds, such as 2-propanone and 2-butanone, in subcutaneous adipose tissue. This is also associated with the abnormal accumulation of phospholipids and ceramides, which correlate strongly with elevated ammonia concentrations in adipose tissue and the generation of oxidation products such as propanal, potentially compromising meat flavor quality.

## 1. Introduction

Tibetan sheep are an indigenous livestock resource unique to the Qinghai–Tibetan Plateau (QTP). They serve not only as an essential means of subsistence for local herdsmen but also as a primary source of economic income [1]. However, the natural environment of the QTP is characterized by harsh conditions, including an average altitude exceeding 4000 m, severe cold, substantial diurnal temperature variations, a short forage growing season, and an unbalanced nutritional supply [2]. Furthermore, the cold season extends for 7–8 months, during which forage becomes withered and scarce. Consequently, with a traditional grazing method, Tibetan sheep struggle to obtain sufficient dietary energy and protein intake during this period [3]. These extreme conditions lead to chronic seasonal nutritional stress, resulting in growth retardation, severe weight loss, extended production cycles, and impaired reproductive performance—all of which severely constrain the sustainable development of the Tibetan sheep industry [4]. Thus, it is necessary to alter the feeding and management approaches of Tibetan sheep to alleviate the body weight loss in the cold season.

To overcome the limitations of grazing and ensure a balanced year-round nutrient supply, indoor and semi-confinement rearing models are increasingly emerging as critical developmental strategies for Tibetan sheep production [5]. Under intensive production conditions, dietary protein must be sufficient to meet the requirements for rapid growth and fattening of Tibetan sheep. Previous studies indicate that during the cold season on the Qinghai–Tibet Plateau, the dietary crude protein level for fattening Tibetan sheep raised indoors should typically be maintained between 12% and 14% [6]. Soybean meal (SBM), the dominant protein source in China’s feed industry, is heavily dependent on imports and therefore highly susceptible to fluctuations in global market prices [7]. In recent years, rising SBM prices led to a persistent shortage in their supply relative to demand [8]. Consequently, exploring inexpensive, efficient, and sustainable alternative protein sources to partially or fully replace SBM has become a critical priority to alleviate the shortage of feed protein resources and ensure stable livestock production [9]. Actually, non-protein nitrogen (NPN) has become a better protein feed substitute for ruminants due to its low price.

In ruminants, rumen microbiota possess a unique ability to convert NPN sources into microbial protein, enabling the partial substitution of traditional protein feeds with appropriate NPN supplementation. This strategy can effectively reduce feed costs while maintaining production performance [10]. NH_4_Cl, a kind of NPN feed additive, serves as an essential nitrogen source for rumen microorganisms, thereby promoting fiber degradation and animal growth when basal dietary true protein is deficient [9]. Furthermore, unlike other NPN sources such as urea or ammonium sulfate, NH_4_Cl is not only cost-effective but also metabolically acidifies body fluids and urine, thereby significantly reducing the risk of urolithiasis [11]. This function is particularly critical for Tibetan sheep raised under indoor feeding conditions, where restricted physical activity often predisposes animals to urine concentration. However, it is important to note that if total dietary nitrogen is already sufficient, additional NH_4_Cl supplementation can cause the rate of ruminal ammonia production to rapidly exceed the microbial utilization capacity. Consequently, excess ammonia enters the bloodstream, potentially inducing ammonia toxicity [12]. Exploring the appropriate addition amount of ammonium chloride is of great practical significance for the rational use of non-protein nitrogen in Tibetan sheep production.

Tibetan sheep meat is characterized by high protein and low fat content [13]. In addition, it possesses a balanced fatty acid profile, marked by a high proportion of unsaturated fatty acids—especially n-3 polyunsaturated fatty acids (n-3 PUFAs)—and is rich in essential and sulfur-containing amino acids [14]. These attributes together confer exceptional nutritional value and health benefits. Among these superior qualities, meat quality serves as a core determinant of market acceptance and consumer eating experience [15]. Among the numerous factors determining the quality of sheep meat, adipose tissue, particularly subcutaneous adipose tissue (SAT), plays a pivotal role. Accounting for more than 70% of total body fat, SAT serves not only as an energy reserve [16] but also as a source of precursors and a carrier for flavor compounds [17]. The characteristic flavor of sheep meat primarily arises from volatile flavor compounds, such as aldehydes, ketones, alcohols, and hydrocarbons, generated through the Maillard reaction and lipid oxidation during thermal processing [18]. Consequently, the extent of fat deposition, fatty acid composition, and overall lipid profile in SAT are key determinants of important sensory attributes in the meat, such as juiciness, tenderness, and flavor intensity [19]. However, the lipid metabolic processes within SAT, which are critical for flavor formation, are highly susceptible to dietary nutritional manipulation. Previous studies have indicated that under conditions of excessive or insufficient dietary nitrogen, supplementation with NH_4_Cl can lead to a significant elevation in rumen ammonia concentrations. The subsequent absorption of excess ammonia across the rumen wall into the systemic circulation can induce oxidative stress in muscle tissues, thereby impairing normal muscle fiber development and deteriorating meat quality [20,21]. Concurrently, ammonia accumulation may disrupt lipid metabolic homeostasis, alter fat deposition patterns, and heighten the susceptibility of unsaturated fatty acids to oxidation, ultimately compromising both the sensory attributes and nutritional value of the meat [22].

In this context, NH_4_Cl has emerged as an effective strategy for reducing feeding costs in the current indoor feeding systems of Tibetan sheep, attributed to its dual functionality as an NPN source and a urinary acidifier for the prevention of urolithiasis. However, the mechanisms by which different levels of NH_4_Cl supplementation affect meat quality, particularly adipose tissue, which serves as the primary source of flavor precursors, remain largely unclear. Currently, the specific mechanisms by which NH_4_Cl regulates lipid metabolism and subsequently influences flavor formation in the subcutaneous adipose tissue of Tibetan sheep remain largely unexplored. To address this gap, this study integrated lipidomics with gas chromatography–ion mobility spectrometry (GC-IMS) to characterize the lipid profiles and volatile compounds in sheep fed graded levels of NH_4_Cl. The objective was to elucidate the lipid metabolic mechanisms underlying the modulation of meat flavor. The findings will provide a theoretical basis for enhancing meat quality and product value through targeted nutritional strategies and offer empirical evidence supporting the scientific and safe application of NH_4_Cl in ruminant production.

## 2. Materials and Methods

### 2.1. Experimental Design and Feeding Management

This study was conducted at the Jinzang Ecological Highland Tibetan Sheep Breeding Professional Cooperative in Haiyan County, Haibei Prefecture, Qinghai Province. Eighty healthy, early-weaned Tibetan lambs (aged 2 months) with similar initial body weights (16.22 ± 1.01 kg) were randomly assigned to four treatment groups. Each group consisted of four replicates, with five lambs per replicate. The graded supplementation levels of NH_4_Cl were established in strict accordance with the Code of Practice for Safe Use of Feed Additives (Announcement No. 2625 of the Ministry of Agriculture and Rural Affairs, MARA). This regulation stipulates that the crude protein (CP) equivalent derived from NPN in the total diet must not exceed 30% of the total CP content. Accordingly, the NH_4_Cl-supplemented levels of the experimental groups were set as follows: 0% (N0, control), 1.49% (N1), 2.24% (N2), and 3.01% (N3). These levels provided 0%, 18.27%, 27.42%, and 36.57% CP of the total CP in the diets, respectively. Based on calculations, the dosages for the N1 and N2 groups fell within the regulatory compliance limits (below the 30% threshold). In contrast, the N3 group was designated as a “marginally supra-optimal” level, designed to evaluate the biological effects of supplementation slightly exceeding the safety threshold. The dietary ingredients and nutrient compositions are presented in Table 1.

Before the commencement of the experiment, all lambs were immunized with a lamb quadruple inactivated vaccine (against clostridial diseases), Peste des Petits Ruminants (PPR) live vaccine, Foot-and-Mouth Disease (FMD) inactivated vaccine, and lamb pox live vaccine. Each lamb was uniquely tagged. The study lasted for 105 days, including a 15-day adaptation period followed by a 90-day experimental period. During the trial, the lambs were fed twice daily at 09:00 and 16:00 and had ad libitum access to both feed and water.

### 2.2. Sample Collection

At the end of the feeding trial, five lambs were randomly selected from each group for slaughter. Feed and water were withdrawn for 12 h before slaughter. After slaughter, dorsal SAT samples were collected from the area between the 12th and 13th ribs. For morphological analysis, a slice of the adipose tissue was rinsed with physiological saline, cut into approximately 1 cm × 1 cm × 1 cm blocks, and fixed in a 4% paraformaldehyde solution. For fatty acid profiling, flavor compound analysis, and lipidomic assays, additional adipose tissue samples were placed in cryotubes, snap-frozen in liquid nitrogen, and stored at −80 °C until analysis.

### 2.3. Measurement Indices and Methods

#### 2.3.1. Histological Analysis of Subcutaneous Adipose Tissue

Following fixation in 4% paraformaldehyde, SAT samples were trimmed, washed, dehydrated, cleared, and embedded in paraffin. Serial sections with a thickness of 4 μm were prepared, stained with hematoxylin and eosin (HE), and mounted for morphological examination using an optical microscope (Olympus Corporation, Tokyo, Japan). The tissue sections were examined under an optical microscope, and images were captured at a magnification of 10×. The actual physical dimensions of each field of view (FOV) were 2236 × 1304 μm. The selection of FOVs adhered to specific criteria to ensure representativeness: orderly cell arrangement, distinct and visible outlines, and an absence of blurring, void spaces, or overlapping cells. Image processing and morphometric analysis were performed using ImageJ software (Version 2.13.0; National Institutes of Health, Bethesda, MD, USA). Measurements were performed in triplicate for each FOV, and the mean value was calculated to represent the experimental data for the corresponding section. All measurements were expressed in micrometers (μm).

#### 2.3.2. Analysis of Fatty Acid Composition in Subcutaneous Adipose Tissue

Fatty acid composition was analyzed following a modified method of Zhang et al. [23]. Lipid extraction was performed first. Briefly, 50 mg of adipose tissue was weighed, and lipids were extracted using a chloroform–methanol mixture (2:1, *v*/*v*). The extracted lipids were then methylated with a sulfuric acid–methanol solution for 30 min. The mixture was then extracted with n-hexane and washed with 5 mL of ultrapure water containing 25 μL of methyl salicylate as the internal standard. The resulting supernatant was collected for chromatographic analysis. Chromatographic separation was performed on an Agilent 19091S-433UI capillary column. The oven temperature was initially held at 40 °C for 5 min, increased to 220 °C at 10 °C/min, and then held at 220 °C for an additional 5 min. Hydrogen served as the carrier gas at a flow rate of 1.0 mL/min. Mass spectrometry detection was conducted using an Agilent 5977B MSD mass spectrometer (Agilent Technologies, Santa Clara, CA, USA).

#### 2.3.3. Lipidomics Analysis of Subcutaneous Adipose Tissue

Lipid extraction and liquid chromatography–tandem mass spectrometry (LC-MS/MS) analysis were primarily performed following the method described by Sa et al. [24]. Mass spectrometry-grade methanol, acetonitrile, and high-performance liquid chromatography (HPLC)-grade isopropanol (Thermo Fisher Scientific, Waltham, MA, USA), and HPLC-grade formic acid and ammonium formate (Sigma-Aldrich, St. Louis, MO, USA)) were used. Precisely weighed samples were spiked with internal standards and extracted using a methyl tert-butyl ether/methanol/water system (8:2.4:2, *v*/*v*/*v*). The internal standards are listed in Table S1. After vortexing, sonication, and centrifugation, the organic phase was collected and dried under a nitrogen stream. The residue was reconstituted in 90% isopropanol/acetonitrile before LC–MS/MS analysis. Chromatographic conditions: Lipid separation was achieved on a C18 column maintained at 40 °C, a flow rate of 300 μL/min, and the sample chamber temperature of 10 °C. Mobile phase A consisted of an aqueous solution containing 10 mmol/L ammonium acetate, while mobile phase B contained 10 mmol/L ammonium acetate in isopropanol/acetonitrile (9:1, *v*/*v*). The gradient elution program was as follows: 0–2 min, 30% B; 2–25 min, B linear increase from 30% to 100%; 25–35 min, re-equilibration at 30% B. Mass spectrometry conditions: An electrospray ionization (ESI) source was operated in positive/negative switching mode. The parameters were set as follows: ion source temperature 300 °C, sheath gas flow 45 arb, auxiliary gas flow 15 arb, spray voltage ±3.0 kV, and capillary temperature 350 °C. Full MS scans were acquired at a resolution of 70,000 (*m*/*z* 200–1800) using data-dependent acquisition (dd-MS^2^) mode, with the top 10 most intense ions selected for higher-energy collisional dissociation (HCD) fragmentation at a resolution of 17,500. To ensure data reliability, a quality control (QC) sample, prepared by pooling equal aliquots of all experimental samples, was inserted after every 10 analytical samples to monitor instrument stability. The acceptance criteria were set as follows: the coefficient of variation (CV) for the peak areas of target lipids was ≤15%, and the CV for internal standard peak areas was ≤10%. Principal component analysis (PCA) was employed to verify the clustering of QC samples. In cases where QC samples exhibited a significant deviation from the cluster center, the corresponding analytical batch was either re-injected or subjected to batch effect correction.

#### 2.3.4. GC-IMS Analysis

A 1.0 mL sample of SAT was accurately transferred into a 20 mL headspace vial and incubated for 15 min before injection. All analyses were performed in triplicate. The headspace sampling parameters were as follows: incubation temperature 50 °C, incubation time 20 min, injection volume 500 µL, splitless injection mode, agitation speed 500 rpm, and syringe temperature 85 °C. GC conditions: The column temperature was maintained at 40 °C. High-purity nitrogen (≥99.999%) was used as the carrier gas. The flow rate was programmed as follows: held at 2.0 mL/min for 2 min, increased linearly to 10.0 mL/min over 8 min, and then increased to 100.0 mL/min over the next 10 min. The total chromatographic run time was 20 min, and the injector temperature was set at 80 °C.

IMS conditions: The IMS detector was equipped with a tritium (3H) ionization source. The drift tube (53 mm) operated at an electric field strength of 500 V/cm and a temperature of 45 °C. High-purity nitrogen (≥99.999%) was used as the drift gas at a flow rate of 150 mL/min. Analyses were performed in positive ion mode.

Qualitative analysis: A standard mixture of six ketones was analyzed to establish a calibration curve relating retention times to retention indices (RI). The RIs of target compounds were calculated based on their retention times. Compound identification was achieved by matching calculated RIs and drift times against the NIST 2020 GC retention index database and the IMS drift time database embedded in the VOCal software 0.4.03 (LECO Corporation, St. Joseph, MI, USA). Identification Strategy and Confidence Levels: Ketones were positively identified by comparing their retention indices (RIs) and drift times (DTs) with those of authentic standards. The remaining volatile compounds were putatively identified by matching their RI and DT values against the NIST 2020 database, with a similarity threshold set at >80%.

Data visualization: The Gallery Plot plugin in the VOCal data processing software was used to generate fingerprints of the volatile components, facilitating the comparison of volatile organic compounds (VOCs) across different samples.

### 2.4. Statistical Analysis

Prior to parametric testing, the normality of the data distribution for all experimental groups was assessed using the Shapiro–Wilk test. The results indicated that the data in all groups followed a normal distribution (*p* > 0.05), thereby satisfying the assumptions required for analysis of variance (ANOVA). Data were analyzed using one-way analysis of variance (ANOVA) in SPSS 27.0. Differences were considered statistically significant at *p* < 0.05. For lipidomics data, orthogonal partial least squares discriminant analysis (OPLS-DA) was performed using SIMCA 14.1. Differential lipid molecules were identified based on variable importance in projection (VIP) scores in combination with Student’s *t*-test, with thresholds set at VIP > 1 and a false discovery rate (FDR) < 0.05. Flavor components were analyzed using the instrument’s built-in software. Additionally, Pearson correlation analysis was conducted to evaluate the relationships between variables, with statistical significance defined as *p* < 0.01 and an absolute correlation coefficient |r| ≥ 0.80 (a stricter threshold is used to reduce the false-positive rate and retain only strong correlations).

## 3. Results

### 3.1. Effects of Dietary Ammonium Chloride Supplementation on the Morphology of Subcutaneous Adipose Tissue in Tibetan Sheep

**Figure 1:**
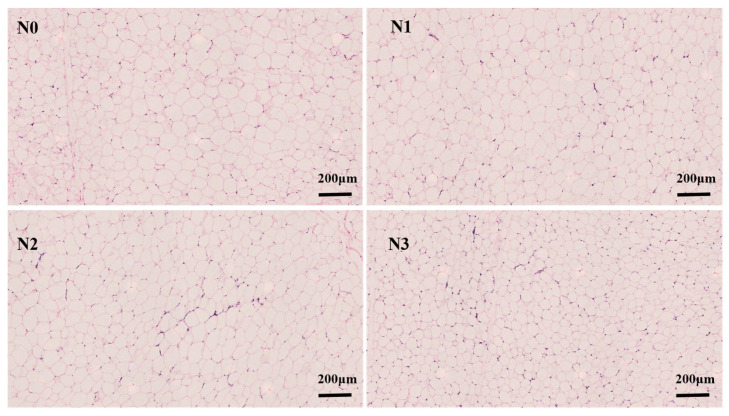
Subcutaneous adipose tissue morphology.

As illustrated in Figure 1 and Table 2, the SAT of Tibetan sheep exhibited a typical honeycomb-like or reticular architecture. The adipocytes were closely packed, polygonal or sub-spherical in shape, with intact morphology and distinct boundaries. These cells were separated by thin connective tissue septa, forming a relatively regular lobular structure. With increasing dietary ammonium chloride (NH_4_Cl) supplementation, a decrease in adipocyte diameter accompanied by an increase in adipocyte density was observed. Specifically, compared with the N0 group, the adipocyte diameter in the N3 group decreased by 10.8%, whereas the adipocyte density increased by 29.7%.

### 3.2. Effects of Dietary Ammonium Chloride Supplementation on Fatty Acid Content in Subcutaneous Adipose Tissue of Tibetan Sheep

As shown in Table 3, compared with the N0 group, the contents of most unsaturated fatty acids, including C19:1n7t, C19:1n10t, C20:1, α-linolenic acid (C18:3n3), C20:2, and C22:1, and n-3 polyunsaturated fatty acids (n-3 PUFAs) were significantly decreased in the N1, N2, and N3 groups (*p* ˂ 0.05). The total saturated fatty acid (total SFA) content in the N3 group was significantly higher compared to the other groups (*p* ˂ 0.05). Conversely, the total monounsaturated fatty acid (total MUFA) content in the N3 group was significantly lower compared to the N1 and N2 groups (*p* ˂ 0.05), while the total polyunsaturated fatty acid (total PUFA) content was significantly lower compared to the N0 group (*p* ˂ 0.05). 

### 3.3. Effects of Dietary Ammonium Chloride Supplementation on Lipid Composition in Subcutaneous Adipose Tissue of Tibetan Sheep

#### 3.3.1. Analysis of Lipid Composition

**Figure 2:**
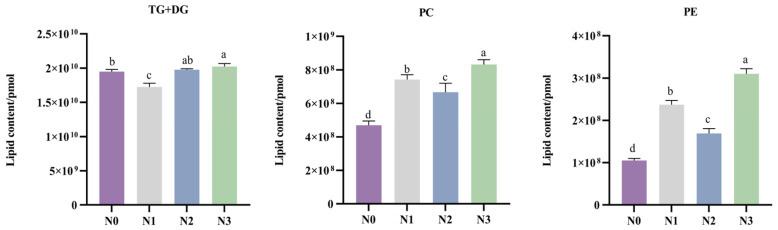
Absolute contents of the major lipid subclasses in subcutaneous adipose tissue of Tibetan sheep. Different letters above the bas indicate significant differences among groups (*p* < 0.05).

A total of 2583 lipid molecules, belonging to 24 lipid subclasses, were identified in the SAT of Tibetan sheep. As shown in Figures S1 and S2 in the Supplementary Material, the predominant lipid subclasses were triglycerides (TGs), phosphatidylcholines (PCs), phosphatidylethanolamines (PEs), and diglycerides (DGs). As shown in Figure 2, the absolute contents of PCs, PEs, and the sum of TGs and DGs in the N3 group were significantly higher than those in the other groups (*p* < 0.05); the absolute contents of PCs and PEs in the N0 group were significantly lower than those in the other groups (*p* < 0.05).

#### 3.3.2. Multivariate Statistical Analysis of Lipid Composition

**Figure 3:**
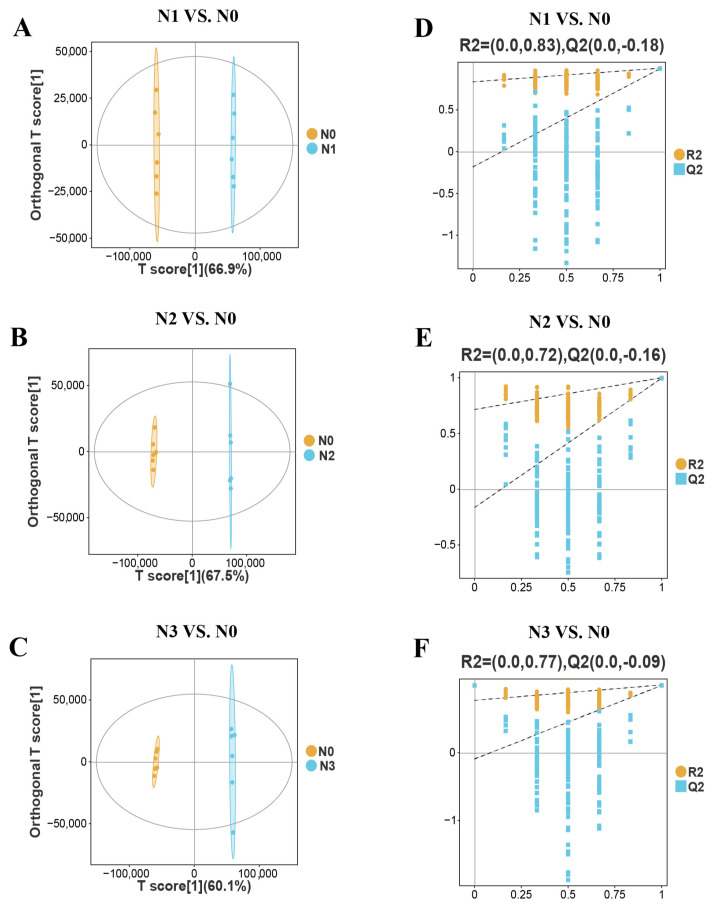
Score plot and permutation test of OPLS-DA. (**A**–**C**) OPLS-DA score plot. The ellipses represent the Hotelling’s T^2^ 95% confidence regions, which visualize the distribution and clustering of samples within each group. (**D**–**F**) Permutation test of the OPLS-DA model. The dashed lines represent the regression fits for R^2^ (model explained variance, in orange) and Q^2^ (model predictive ability, in blue). The horizontal dashed line indicates the expected Q^2^ value of a random model.

To enhance group discrimination, orthogonal partial least squares discriminant analysis (OPLS-DA) was performed on the lipidomics data. As illustrated in Figure 3, the N1, N2, and N3 groups were distinctly separated from the N0 group, clustering on opposite sides of the score plots, indicating strong discriminatory power of the model. Permutation tests were conducted to evaluate the robustness and predictive capability of the model. As shown in Figure 3, the R^2^Y values for the N1 vs. N0, N2 vs. N0, and N3 vs. N0 models were 0.83, 0.72, and 0.77, respectively, indicating that the models possessed high explanatory power for the training data. Furthermore, all R^2^Y intercepts were 0.0, and the Q^2^ intercepts were −0.18, −0.16, and −0.09, respectively. Since all Q^2^ intercepts were less than zero, this suggests that the models were not overfitted and possessed good predictive reliability [25].

#### 3.3.3. Analysis of Differential Lipids in Subcutaneous Adipose Tissue

**Figure 4:**
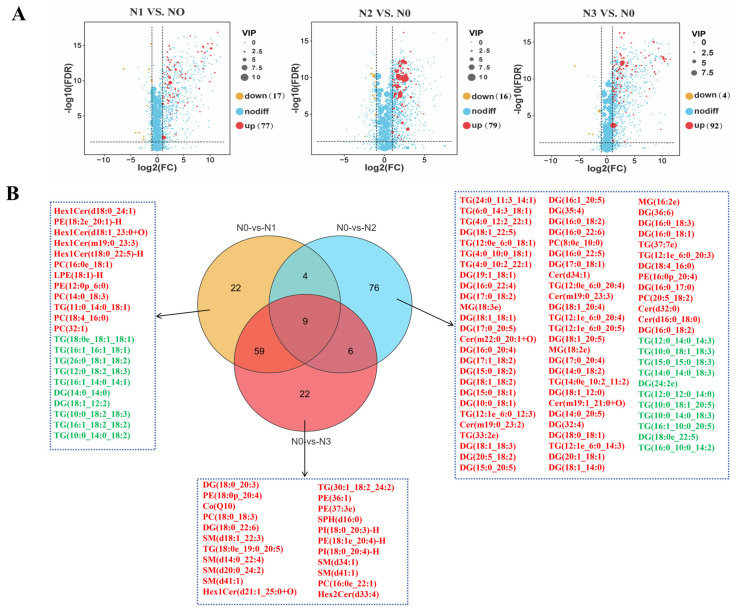
Differential lipid analysis: (**A**) Volcano plot of differential lipids. Vertical dashed line: log_2_(FC) = 0, distinguishing up-regulated genes (log_2_FC > 0, red), down-regulated genes (log_2_FC < 0, yellow), and non-differentially expressed genes (log_2_FC ≈ 0, blue). Horizontal dashed line: significance threshold (-log_10_(FDR) = 1, FDR = 0.1), defining the statistical significance of differentially expressed genes. Dot size represents VIP value; color corresponds to expression trend; numbers in parentheses indicate the count of genes for each trend. (**B**) Venn diagram of differential lipids (red: upregulated; green: downregulated). PC, phosphatidylcholine; PE, phosphatidylethanolamine; LPE, lysophosphatidylethanolamine; PI, phosphatidylinositol; SM, sphingomyelin; SPH, sphingosine; Hex1Cer, hexosyl ceramide; Hex2Cer, hexosyl ceramide; Cer, ceramides; MG, monoglyceride; DG, diglyceride; TG, triglyceride; Co, coenzyme.

Based on the OPLS-DA model, a total of 285 differential lipid metabolites were identified through pairwise group comparisons, using the criteria of Variable Importance in Projection (VIP) ≥ 1.0, |Fold Change| ≥ 2, and Adjusted *p* ˂ 0.05. As shown in Figure 4A, compared with the N0 group, a substantial number of differential lipid molecules were identified in all treatment groups, with the majority being upregulated. Specifically, in the N1 vs. N0 comparison, 94 differential lipids were identified, of which 77 were significantly upregulated, and 17 were significantly downregulated. In the N2 and N0 comparison, 95 differential lipids were identified, including 79 upregulated and 16 downregulated. In the N3 vs. N0 comparison, 96 differential lipids were observed, with 92 significantly upregulated and only 4 downregulated. As illustrated in Figure 4B, the differential lipid molecules were primarily glycerolipids, such as diglycerides (DGs) and triglycerides (TGs), and glycerophospholipids, such as phosphatidylcholines (PCs) and phosphatidylethanolamines (PEs).

### 3.4. Effects of Dietary Ammonium Chloride Supplementation on Volatile Flavor Compounds in Subcutaneous Adipose Tissue of Tibetan Sheep

**Figure 5:**
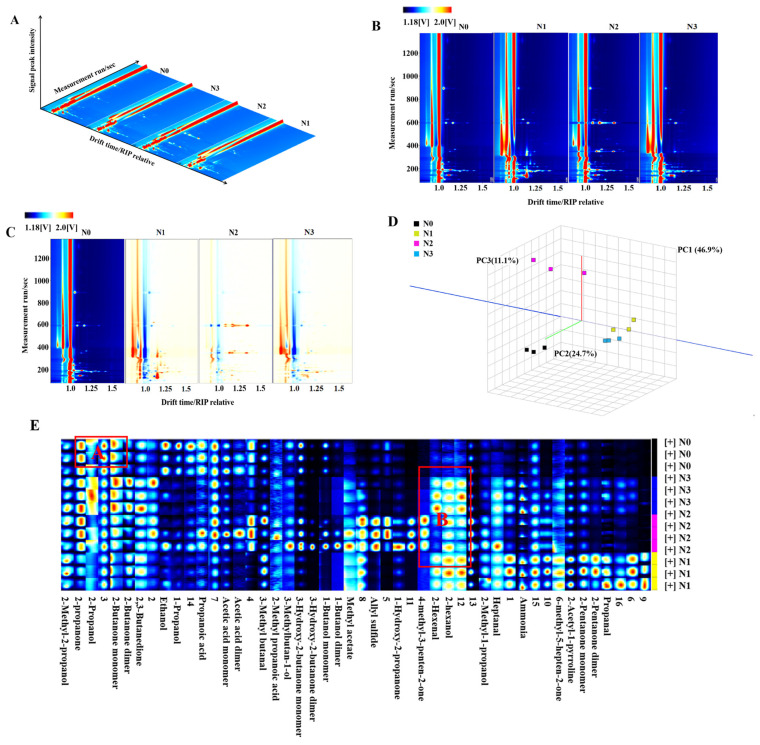
GC-IMS analysis of volatile flavor compounds in the subcutaneous fat of Tibetan sheep: (**A**) 3D topographic map. (**B**) 2D topographic map, where the blue background and red vertical line at abscissa 1.0 represent the baseline and reactive ion peak (RIP), respectively, and each point represents a single volatile compound (color depth indicates peak intensity). (**C**) Difference map, with N0 group as the reference (white = no difference, red = higher content in target group, blue = lower content). (**D**) PCA score plot, the scatter plot shows the distribution of samples across the first three principal components: PC1 (46.9%), PC2 (24.7%), and PC3 (11.1%), which cumulatively explain 82.7% of the total variance. (**E**) Fingerprint plot, where each row = one sample (N0–N3); each column = one volatile compound. Color gradient (blue → red → white) indicates peak intensity (low → high). Red boxes mark characteristic regions: A = N0-specific compounds; B = N1/N2/N3-enriched compounds.

The GC-IMS analysis spectra of volatile flavor compounds in the SAT of Tibetan sheep are presented in Figure 5. As shown in Figure 5A, all groups exhibited high overall similarity, indicating that their basic flavor profiles were largely comparable. However, distinct differences in signal intensity were observed in specific regions, reflecting variations in the types and concentrations of flavor compounds among the groups. Notably, the N2 group displayed a greater number of signal peaks with higher characteristic peak intensities. Figure 5B displays the two-dimensional topographic plot, where the background is blue, and the red vertical line at an abscissa of 1.0 represents the reactive ion peak (RIP). Each point adjacent to the RIP represents a distinct volatile organic compound (VOC). The color in the GC–IMS spectra represents the peak intensity of each compound, with deeper colors (shifting from blue to red) indicating higher peak intensity [26]. The VOC profiles of the N1 and N3 groups were similar, whereas the N2 group exhibited greater diversity and higher VOC content.

To further intuitively compare the differences in volatile components, the spectrum of the N0 group was used as a reference, and the spectra of the other groups were subtracted from it to generate the difference plot shown in Figure 5C. In this plot, a white background indicates that the content of a specific VOC is identical between the target group and the N0 group. Red indicates a higher concentration of the substance in the target sample compared with the reference, while blue indicates a lower concentration. As shown in Figure 5C, the N3 group showed fewer red and blue signals with lighter coloration, indicating that its volatile profile was more similar to that of the N0 group. Conversely, the N2 and N1 groups exhibited more red signals with deeper coloration, indicating greater diversity and higher concentrations of volatile substances. The principal component analysis (PCA) of the volatile flavor compounds is shown in Figure 5D. Clear differences were observed in the volatile compound profile among the groups. The contribution rates of the first three principal components were 46.9%, 24.7%, and 11.1%, respectively. The clear separation among the groups indicates significant differences in their volatile substance profiles. As indicated by the fingerprint spectra in Figure 5E, the types of volatile substances in the N1 and N3 groups were relatively similar, whereas the N2 group exhibited the greatest diversity of volatile compounds. Region A highlights the predominant volatile substances identified in the N0 group, including acetone (2-propanone), 2-propanol, and 2-butanone monomer. Region B corresponds to the major volatile substances in the N1, N2, and N3 groups, including trans-2-hexenal and 2-hexanol.

As presented in Table 4, the contents of most aroma-active volatile compounds, such as 2-propanone, ethanol, acetic acid monomer, 2-butanone monomer, and 1-propanol, were significantly higher in the N0 group compared to the other three groups (*p* ˂ 0.05). The relative content of ammonia in the N1 and N3 groups was significantly higher compared to the N0 and N2 groups (*p* ˂ 0.05). Additionally, the content of allyl sulfide in the N2 group was significantly higher compared to the other three groups (*p* ˂ 0.05).

### 3.5. Correlation Analysis Between Key Volatile Flavor Compounds and Differential Lipid Molecules in Subcutaneous Adipose Tissue

**Figure 6:**
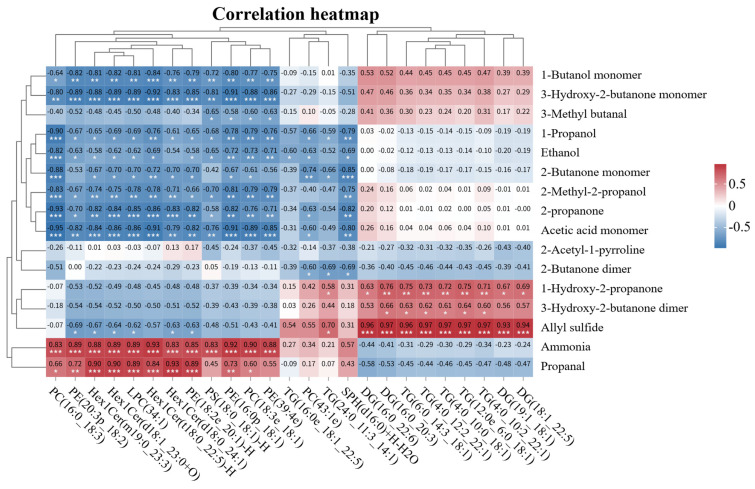
Correlation heatmap between key volatile flavor compounds and differential lipid molecules in the subcutaneous fat of Tibetan sheep. Significance levels are indicated as follows: no mark, *p* ≥ 0.05; *, 0.01 < *p* < 0.05; **, 0.001 < *p* ≤ 0.01; ***, *p* ≤ 0.001.

Based on the screening criteria of VIP ≥ 1.0, |Fold change| ≥ 2, and adjusted *p* < 0.05, nine differential lipids common to all four groups were selected. Additionally, using criteria of VIP ≥ 1.0, |Fold change| ≥ 8, and adjusted *p* < 0.05, 16 differential lipids unique to the NH_4_Cl-supplemented groups were screened, resulting in a total of 25 core differential lipids selected to conduct correlation analysis. As shown in Figure 6, the majority of TG and DG differential lipid molecules, including TG (6:0_14:3_18:1), TG (4:0_12:2_22:1), and DG (16:0_20:3), exhibited significant positive correlations with allyl sulfide (r = 0.93–0.97, *p* < 0.01). Most differential lipid molecules belonging to the PC, PE, and Hex1Cer classes, such as PC (16:0_18:3), PE (20:3p_18:2), and Hex1Cer(m19:0_23:3), showed significant positive correlations with ammonia (r = 0.83–0.93, *p* < 0.01). All Hex1Cer differential lipid molecules were significantly positively correlated with propanal (r = 0.84–0.93, *p* < 0.01). Conversely, PC (16:0_18:3) displayed significant negative correlations with 3-hydroxy-2-butanone monomer, 1-propanol, ethanol, 2-butanone monomer, 2-methyl-2-propanol, 2-propanone, and acetic acid monomer (r = −0.95 to −0.80, *p* < 0.01). Furthermore, the majority of PC, PE, and Hex1Cer differential lipid molecules exhibited significant negative correlations with 1-butanol monomer, 3-hydroxy-2-butanone monomer, 2-propanone, and acetic acid monomer (r = −0.95 to −0.80, *p* < 0.01).

## 4. Discussion

Various nutritional factors regulate the morphological structure and functional characteristics of adipose tissue, a major energy storage depot in animals. The size, density, and organization of adipocytes are directly correlated with the distribution patterns and deposition efficiency of adipose tissue [27]. In the present study, dietary ammonium chloride supplementation decreased the diameter and increased the density of subcutaneous adipocytes in Tibetan sheep. Although these changes were not statistically significant, they reflect a potential regulatory effect of ammonium chloride on adipocyte morphology. These findings are in agreement with the reported effects of ammonium chloride on intermuscular fat development. Previous research indicated that low doses of ammonium chloride may influence adipocyte proliferation through activation of the AMPK pathway. In contrast, the morphological changes observed at higher supplementation might relate to the alterations in the overall metabolic environment [28]. The impact of different ammonium chloride supplementation on SAT morphology appeared to be dose-dependent. Among all groups, the N3 group exhibited the smallest cell diameter and the highest cell density, suggesting that high dosage correlates with significant adipocyte morphological changes. This effect might be associated with disturbances in systemic acid–base balance following ammonium chloride supplementation. The accumulation of excess ammonia might be linked to intracellular pH dysregulation and oxidative stress, potentially contributing to impaired cell membrane integrity and inhibiting normal cell growth [29]. Additionally, elevated rumen ammonia nitrogen levels in ruminants might promote the conversion of acetate to fatty acids. This indirectly alters lipid deposition patterns in adipocytes and contributes to morphological changes [30]. The effects of ammonium chloride on subcutaneous adipocyte morphology in this study were not statistically significant. This contrasts with the significant abnormalities, like cellular disarray and membrane rupture, reported previously in intermuscular fat [31]. This discrepancy might also reflect tissue specificity. As a major energy reserve with relatively lower metabolic activity, SAT may exhibit greater tolerance to the metabolic changes induced by ammonium chloride [32]. In conclusion, dietary ammonium chloride supplementation had a dose-dependent effect on SAT morphology in Tibetan sheep by indirectly altering the lipid metabolic environment and influencing the cellular physiological state. However, SAT appeared to exhibit greater tolerance to ammonium chloride compared to intermuscular adipose tissue, as no significant morphological abnormalities were observed. This provides a structural basis for Tibetan sheep to maintain metabolic balance under this nutritional intervention strategy.

The fatty acid composition of SAT is a critical determinant of the flavor, tenderness, and nutritional value of Tibetan sheep meat. Monounsaturated fatty acids (MUFAs) are strongly associated with improved meat palatability, while n-3 polyunsaturated fatty acids (n-3 PUFA) can help reduce the risk of cardiovascular disease in humans [33]. Additionally, the n-6/n-3 PUFA ratio is widely recognized as a vital criterion for evaluating the health value of meat products [33]. In this study, increasing dietary ammonium chloride supplementation levels was found to significantly decrease the contents of most unsaturated fatty acids and n-3 PUFAs in SAT, while the total SFA content significantly increased in the N3 group. These findings suggest that ammonium chloride may be associated with the synthesis and accumulation of major lipid components in subcutaneous fat, possibly linked to changes in rumen fermentation patterns and systemic metabolic processes [33]. Firstly, as an acidic salt, ammonium chloride dissociates into ammonia and chloride ions upon entering the rumen, thereby lowering rumen pH and altering the structure and metabolic activity of the rumen microbial community [34]. Previous studies have shown that low rumen pH inhibits the biohydrogenation of certain unsaturated fatty acids [35]. However, in the present study, the content of n-3 PUFAs decreased. This may be due to reduced fatty acid absorption efficiency or changes in their redistribution within the body, caused by ammonium chloride-induced changes in the rumen environment. The significant increase in total SFA content in the N3 group suggests that high levels of ammonium chloride may promote the synthesis of saturated fatty acids or reduce their oxidative utilization, thereby enhancing SFA deposition in adipose tissue. Secondly, the total MUFA content in the N3 group was significantly lower compared to the N1 and N2 groups, indicating that high levels of ammonium chloride might inhibit MUFA synthesis or facilitate their conversion into SFA. Given that MUFAs enhance meat flavor and palatability, the observed decrease could negatively affect the quality of Tibetan sheep meat [36]. Additionally, the total PUFA content in the N3 group was significantly lower than that in the N0 group. This suggests that high ammonium chloride supplementation decreased PUFA deposition, particularly n-3 PUFAs, likely by affecting lipid metabolism pathways. In conclusion, dietary ammonium chloride supplementation significantly influenced the fatty acid composition of SAT in Tibetan sheep by modulating the rumen environment and lipid metabolic pathways. While moderate supplementation appeared to have a minimal impact on fatty acid composition, high levels significantly reduced the concentrations of unsaturated fatty acids, especially n-3 PUFAs, while increasing the deposition of saturated fatty acids. These alterations may negatively impact both the nutritional value and flavor quality of Tibetan sheep meat.

Lipidomics, as a key branch of systems biology, enables the comprehensive and quantitative investigation of the composition, structure, abundance, and metabolic dynamics of lipid molecules within an organism, tissue, or cell [37]. Within the lipid metabolic system, triglycerides (TGs), formed by the esterification of a glycerol molecule with three fatty acid chains, serve as the core functional components. As the primary form of energy storage, TGs accumulate extensively in adipose tissue [38]. Notably, the unsaturated fatty acid chains enriched in TGs (e.g., 18:1, 18:2, and 18:3) serve as a crucial precursor for the formation of esters and aldehydes via lipid oxidation during heating, thereby contributing to meat flavor development [39]. In this study, a total of 2583 lipid molecules covering 24 lipid subclasses were identified in the SAT of Tibetan sheep. Among these, TGs accounted for the largest proportion of total lipids, reaffirming their role as the primary energy storage form and indicating a robust substrate foundation for the formation of volatile flavor compounds. This study also found that different ammonium chloride supplementation levels significantly modulated the absolute concentrations of major lipid components in the SAT. Specifically, the absolute concentrations of TGs, diglycerides (DGs), phosphatidylcholines (PCs), and phosphatidylethanolamines (PEs) were highest in the N3 group, whereas the combined absolute concentrations of PCs and PEs were lowest in the N0 group. These results suggested that a high level of ammonium chloride supplementation may be associated with the synthesis and accumulation of major lipid components in subcutaneous fat, possibly linked to alterations in rumen fermentation patterns and systemic metabolic processes. As the direct precursor for TG synthesis, the significant increase in DGs in the N3 group likely provided ample substrate supply for the elevated TG synthesis, thereby activating the lipogenic pathways and enhancing fat deposition efficiency [40]. Phospholipids play an irreplaceable role among lipid components associated with cell membrane structure and function. As an essential constituent of cell membranes, changes in their abundance are directly associated with membrane fluidity, signal transduction efficiency, and the overall metabolic state of the cell [41]. In the present study, the absolute concentrations of PCs and PEs were highest in the N3 group, suggesting that high ammonium chloride levels may exert a significant influence on cell membrane remodeling and metabolic regulation in the subcutaneous adipocytes of Tibetan sheep. This alteration in lipid composition corresponds with the morphological characteristics observed in the N3 group, namely, decreased adipocyte diameter and increased cell density. This suggests that a high level of ammonium chloride affects adipocyte physiology at both compositional and structural levels.

Volatile flavor compounds are key determinants of meat quality, as their composition and concentration directly dictate the sensory attributes. The volatile organic compounds (VOCs) identified in this study in the SAT were primarily aldehydes, alcohols, and ketones, consistent with previous findings by Wang F et al. [42]. Aldehydes are key contributors to the characteristic flavor of sheep meat. While most aldehydes contribute pleasant aromas at concentrations below their odor thresholds, excessive concentrations are typically associated with lipid oxidation and rancidity, resulting in off-odors [43]. For example, hexanal imparts subtle apple- or leaf-like notes at low concentrations, but at high concentrations, it disrupts the overall flavor balance by masking other characteristic flavor compounds. This phenomenon is associated with the substantial generation of hexanal during the thermal oxidation of linoleic acid [44]. The fingerprint spectra in this study revealed elevated levels of 2-hexenal in the N1, N2, and N3 groups, suggesting that ammonium chloride supplementation may promote lipid oxidation, potentially leading to excessive aldehyde formation and undesirable flavor development. Ketones generally possess floral, fruity, and creamy aromas and play a significant regulatory role in overall meat flavor [45]. In the present study, acetone and 2-butanone monomers in the N0 group were significantly higher compared to the ammonium chloride-supplemented groups, and their levels decreased with increasing ammonium chloride supplementation. This decline indicates a reduction in beneficial ketone-derived aroma components, thereby directly compromising the flavor richness of the SAT. In addition, the ammonia content in the N1 and N3 groups was significantly higher compared to the N0 and N2 groups, and such ammonia-derived odors may exert a negative impact on the sensory quality of the meat. Meanwhile, significantly higher propanal content in the N1 group and increased allyl sulfide content in the N2 group suggest that low or medium ammonium chloride levels may selectively promote certain characteristic flavor compounds. However, these increases generally fail to compensate for the broader loss of beneficial flavor constituents.

As core precursors of meat flavor compounds, lipids undergo compositional changes that affect volatile flavor compound content. Understanding the correlation between these two factors is crucial for elucidating the mechanisms behind the nutritional regulation of flavor formation [46]. In this study, correlation analysis revealed that the majority of differential lipid molecules exhibited a significant positive correlation with allyl sulfide. Allyl sulfide is an organic sulfur compound characterized by a potent, volatile flavor [47]. The observed positive correlation suggests that these lipids may serve as precursors in the synthesis of allyl sulfide or indirectly promote its accumulation via metabolic regulation. Most differential lipid molecules belonging to the PC, PE, and Hex1Cer classes showed a significant positive correlation with ammonia, while all Hex1Cer differential lipids were significantly positively correlated with propanal. As core components of cell membranes, variations in PC and PE content are closely related to membrane stability and lipid oxidation processes [48]. Excessive ruminal ammonia accumulation associated with NH_4_Cl supplementation may disturb phospholipid metabolism, facilitating ammonia release and the generation of aldehydes such as propanal. Hex1Cer belongs to the sphingolipid class, and its metabolic dysregulation is typically associated with cellular stress responses [49]. The strong correlation between Hex1Cer and propanal suggests a potential link. Based on this, we propose that NH_4_Cl-associated lipid stress could influence aldehyde formation through metabolic pathways involving sphingolipid metabolism. Furthermore, as a characteristic oxidation product of unsaturated fatty acids, the excessive accumulation of propanal may pose a risk of lipid oxidative rancidity [50].

Notably, the phospholipid molecule PC (16:0_18:3) exhibited a strongly significant negative correlation with various beneficial aroma compounds, such as 3-hydroxy-2-butanone monomer, 1-propanol, 2-butanone monomer, and 2-methyl-2-propanol. Similarly, many differential lipid molecules belonging to the PC, PE, and Hex1Cer classes showed significant negative correlations with 1-butanol monomer, 2-propanone, and acetic acid monomer. These aroma compounds are characterized by fresh fruity, creamy, and fruity notes, constituting essential components of the pleasant flavor profile of Tibetan sheep meat [51]. In this study, the contents of beneficial aroma compounds, such as 2-propanone and 2-butanone monomer, were significantly higher in the N0 group than in the NH_4_Cl-supplemented groups. This negative correlation aligns with the observations from the lipid composition analysis, where the high-dose NH_4_Cl group exhibited significantly elevated PC and PE levels but reduced contents of beneficial flavor compounds. This suggests that NH_4_Cl-induced phospholipid accumulation may compromise meat flavor quality by inhibiting the synthesis or accelerating the degradation of beneficial aroma compounds. Furthermore, since NH_4_Cl supplementation reduced unsaturated fatty acid content, it’s speculated that the disruption of phospholipid metabolism and reduction in these fatty acids may be linked to the loss of beneficial flavor compounds through a potential synergistic effect. This suggests a relationship between NH_4_Cl supplementation and flavor deterioration in Tibetan sheep meat.

## 5. Conclusions

The study indicates that dietary NH_4_Cl supplementation levels exceeding 2.24% are associated with alterations in lipid metabolism and reduced synthesis of unsaturated fatty acids and beneficial flavor compounds, such as 2-propanone and 2-butanone, in subcutaneous adipose tissue. This is also associated with the abnormal accumulation of phospholipids and ceramides, which correlate strongly with elevated ammonia concentrations in adipose tissue and the generation of oxidation products such as propanal, potentially compromising meat flavor quality. Based on the analyses of subcutaneous adipose tissue lipids, flavor compounds, and related metabolites, it can be inferred that maintaining dietary NH_4_Cl supplementation below 2.24% may help balance the physiological need for urinary calculi prevention while preserving meat quality in Tibetan sheep. It should be noted that this inference is limited by the scope of the study; since indices related to animal performance, sensory attributes of meat products, and direct physiological endpoints were not assessed, this proposal cannot be regarded as a definitive feeding guideline. Further investigations incorporating these parameters are required to validate the practical applicability of this supplementation threshold.

## Figures and Tables

**Table 1:** Dietary ingredients and nutritional composition (dry matter basis, %).

Items	Treatment			
	N0 Group	N1 Group	N2 Group	N3 Group
Oat hay	15.00	15.00	15.00	15.00
Oat silage	15.00	15.00	15.00	15.00
Corn	38.30	37.36	41.30	49.63
Soybean meal	3.20	1.40	0.77	0.70
Rapeseed meal	13.20	5.81	4.90	4.27
Cotton meal	3.11	1.75	0.70	0.70
Palm meal	7.50	17.5	15.40	7.00
NH_4_Cl	0	1.49	2.24	3.01
NaCl	0.70	0.70	0.70	0.70
NaHCO_3_	0.07	0.07	0.07	0.07
Stone powder	0.70	0.70	0.70	0.70
4%Premix ^1^	2.80	2.80	2.80	2.80
1%Premix ^2^	0.42	0.42	0.42	0.42
Total	100	100	100	100
Nutrient components ^3^				
DE/(MJ/kg)	11.34	10.87	10.86	11.07
Crude protein	13.35	13.34	13.36	13.46
Ether Extract	2.69	3.11	3.08	2.86
Neutral detergent fiber	44.65	49.67	47.99	42.57
Acidic detergent fiber	25.53	27.13	26.18	23.63
Ash	4.94	4.63	4.40	4.07
Calcium	0.84	0.83	0.82	0.81
Phosphorus	0.17	0.17	0.18	0.20

^1^ The 4% premix provided the following per kg diets: NaHCO_3_ 85 g; CaHPO_4_ 85 g; glucose 500 g; maifanite 300 g; nicotinamide 125 mg. ^2^ The 1% premix provided the following per kg diets: CuSO_4_ 1.5 g, MnSO_4_ 4 g, ZnO 5 g, Na_2_SeO_3_ 1 g; Ca (IO_3_)_2_ 1.5 g; CoSO_4_ 0.5 g; VA 145,000 IU; VD_3_ 34,000 IU; VE 250 mg; antioxidant iron 5 g. ^3^ The digestible energy is a calculated value, while other indexes are measured values.

**Table 2:** Effects of dietary ammonium chloride level on subcutaneous adipose tissue morphology in Tibetan sheep.

Items	Groups				SEM	*p*-Value
	N0	N1	N2	N3		
Cell diameter/um	84.04	81.39	81.77	74.97	2.144	0.541
Cell density (cells)	748.44	884.00	837.44	971.33	43.550	0.369

**Table 3:** Effects of dietary ammonium chloride level on subcutaneous fatty acid content in Tibetan sheep.

Items	Groups				SEM	*p* Value
	N0	N1	N2	N3		
C12:0	49.76 ^ab^	68.39 ^a^	47.38 ^ab^	35.40 ^b^	4.392	0.029
C13:0	2.10	2.19	1.89	1.73	0.125	0.620
C14:0	726.76 ^a^	842.69 ^a^	748.58 ^a^	391.33 ^b^	52.924	<0.001
C14:1	20.36 ^a^	23.69 ^a^	18.22 ^a^	7.15 ^b^	2.201	0.013
C15:0	59.37	64.97	68.11	50.19	3.354	0.265
C15:1T	7.91	4.19	6.49	2.67	1.183	0.454
C16:0	3175.09 ^a^	3638.60 ^a^	3719.24 ^a^	1979.28 ^b^	221.388	<0.001
C16:1T	32.14 ^a^	24.74 ^a^	27.14 ^a^	17.11 ^b^	1.799	0.002
C16:1	331.63 ^ab^	422.78 ^a^	446.36 ^a^	238.72 ^b^	27.717	0.004
C17:0	141.81	140.83	163.06	92.29	11.688	0.171
C17:1T	8.02 ^a^	5.61 ^ab^	5.29 ^b^	4.66 ^b^	0.469	0.025
C17:1	69.36	71.75	80.09	50.78	5.336	0.277
C18:0	2626.46 ^ab^	2824.46 ^a^	3087.11 ^a^	1908.68 ^b^	158.424	0.019
C18:1n9t	155.14 ^a^	144.97 ^a^	119.94 ^ab^	67.01 ^b^	12.313	0.018
C18:1n11t	57.21 ^a^	48.21 ^ab^	41.48 ^ab^	25.58 ^b^	4.300	0.029
C18:1n9c	1403.97 ^b^	3125.14 ^a^	3144.01 ^a^	1959.34 ^ab^	258.868	0.006
C18:1n11c	2462.08 ^a^	1961.99 ^ab^	2188.96 ^a^	1161.01 ^b^	171.390	0.012
C19:1n7t	6.71 ^a^	2.62 ^b^	2.32 ^b^	1.51 ^b^	0.623	<0.001
C19:1n10t	7.75 ^a^	3.85 ^b^	3.53 ^b^	2.76 ^b^	0.619	<0.001
C18:2n6c	214.99	206.64	187.97	145.36	10.533	0.056
C20:0	26.23	25.62	24.54	23.27	1.222	0.878
C20:1T	0.87	0.95	0.85	0.80	0.037	0.600
C18:3n6	2.80	2.85	2.57	2.39	0.102	0.396
C20:1	43.31 ^a^	26.31 ^b^	22.29 ^b^	13.51 ^b^	3.589	0.002
C18:3n3	29.74 ^a^	23.02 ^b^	19.32 ^b^	17.78 ^b^	1.539	0.003
C21:0	2.29	1.79	1.59	1.69	0.153	0.425
C20:2	2.37 ^a^	1.37 ^b^	1.19 ^b^	1.10 ^b^	0.162	<0.001
C22:0	3.41	3.89	3.40	4.13	0.246	0.713
C22:1T	1.20	1.24	1.07	0.89	0.055	0.076
C20:3n6	2.71	2.45	2.21	1.92	0.120	0.092
C22:1	34.01 ^a^	24.40 ^b^	22.26 ^b^	14.50 ^b^	2.318	0.002
C23:0	1.23	1.31	1.25	1.06	0.045	0.234
C20:4n6	6.89	8.80	9.15	5.66	0.586	0.091
C22:2	1.33	1.04	1.13	0.93	0.064	0.141
C24:0	1.13	0.83	0.93	0.79	0.057	0.123
C22:4	1.46	2.13	1.79	0.97	0.172	0.070
C22:5n6	6.29 ^a^	6.95 ^a^	6.70 ^a^	5.24 ^b^	0.228	0.009
Total_SFA	6832.52 ^b^	7630.69 ^b^	7883.44 ^b^	4505.34 ^a^	427.830	<0.001
Total_MUFA	4641.68 ^b^	5892.43 ^a^	6130.30 ^a^	3567.99 ^b^	335.625	0.001
Total_PUFA	268.58 ^a^	255.26 ^ab^	232.03 ^ab^	181.37 ^b^	12.654	0.040
n-3 PUFA	29.74 ^a^	23.02 ^b^	19.32 ^b^	17.78 ^b^	1.539	0.003
n-6 PUFA	238.84	232.24	212.71	163.58	11.500	0.053
n-6/n-3 PUFA	8.02	10.05	11.01	9.39	0.431	0.064

Note: Different letters in the same industry indicate significant differences (*p* < 0.05), otherwise the differences are not significant.

**Table 4:** Effects of dietary ammonium chloride supplementation levels on key volatile compounds in subcutaneous fat of Tibetan sheep.

Items	Odor	Groups				SEM	*p* Value
		N0	N1	N2	N3		
Ammonia	Ammoniacal	29.34 ^b^	67.07 ^a^	42.03 ^b^	67.02 ^a^	5.240	<0.001
2-Propanone	Spicy, caramel-like, fresh	15.36 ^a^	3.015 ^c^	8.893 ^b^	7.679 ^b^	1.364	<0.001
Ethanol	Pungent, alcoholic	6.960 ^a^	1.397 ^b^	2.369 ^ab^	1.164 ^b^	0.834	0.014
Acetic acid monomer	Acidic	6.238 ^a^	1.111 ^c^	3.344 ^b^	1.679 ^c^	0.616	<0.001
3-Hydroxy-2-butanone monomer	Buttery, creamy	4.874 ^a^	0.708 ^b^	3.711 ^a^	0.769 ^b^	0.595	0.001
2-Butanone dimer	Fruity, camphoraceous	2.460 ^a^	1.325 ^b^	1.356 ^b^	2.298 ^a^	0.194	0.028
2-Butanone monomer	Fruity, camphoraceous	2.369 ^a^	0.537 ^c^	1.173 ^b^	1.412 ^b^	0.207	<0.001
Propanal	Aromatic, oily, fruity	1.885 ^b^	10.13 ^a^	1.886 ^b^	4.767 ^b^	1.065	<0.001
3-Methyl butanal	Alcoholic, fruity	1.672	1.316	1.646	0.707	0.186	0.231
2-Methyl-2-propanol	Camphoraceous	1.540 ^a^	0.892 ^b^	1.167 ^ab^	0.926 ^b^	0.093	0.018
1-Butanol monomer	Alcoholic (whiskey), fruity (banana)	1.402 ^a^	0.236 ^b^	1.315 ^ab^	0.248 ^b^	0.200	0.015
1-Propanol	Fresh, alcoholic, leathery	1.234 ^a^	0.190 ^b^	0.413 ^b^	0.221 ^b^	0.140	0.002
3-Hydroxy-2-butanone dimer	Buttery, creamy	1.267	0.163	2.776	0.242	0.495	0.212
2-Acetyl-1-pyrroline	Minty	1.020 ^a^	1.058 ^a^	0.809 ^b^	0.709 ^b^	0.055	0.036
Allyl sulfide	Garlic-like	0.637 ^b^	0.265 ^b^	2.410 ^a^	0.232 ^b^	0.274	<0.001
1-Hydroxy-2-propanone	Buttery, vanilla, spicy, malty	0.438	0.245	1.158	0.205	0.160	0.103

Note: Different letters in the same industry indicate significant differences (*p* < 0.05), otherwise the differences are not significant.

## Data Availability

The original contributions presented in this study are included in the article/Supplementary Materials. Further inquiries can be directed to the corresponding authors.

## Supplementary Materials

The following supporting information can be downloaded at https://www.mdpi.com/article/10.3390/foods15030554/s1, Figure S1: Number of lipid molecules; Figure S2: Pie chart of the relative contents of lipid molecules; Figure S3: Bar chart of the absolute contents of lipid molecules. Table S1: List of internal standards.

## Abbreviations

The following abbreviations are used in this manuscript:

GC–IMS	Gas chromatography–ion mobility spectrometry
GC–MS	Gas chromatography–mass spectrometry
SFA	Saturated fatty acid
PUFA	Polyunsaturated fatty acid
MUFA	Monounsaturated fatty acid
OPLS-DA	Orthogonal partial least squares discriminant analysis
