# Supplementary material for: Effects of Dietary Ammonium Chloride Supplementation on the Lipidome and Volatile Flavor Compounds in the Subcutaneous Adipose Tissue of Tibetan Sheep
Source: Foods. 2026 Feb 4;15(3):554. doi: 10.3390/foods15030554 (PMC12896583; doi:10.3390/foods15030554)
Supplement: Supplementary file 1 [file foods-15-00554-s001.zip › foods-4072664-supplementary.pdf]

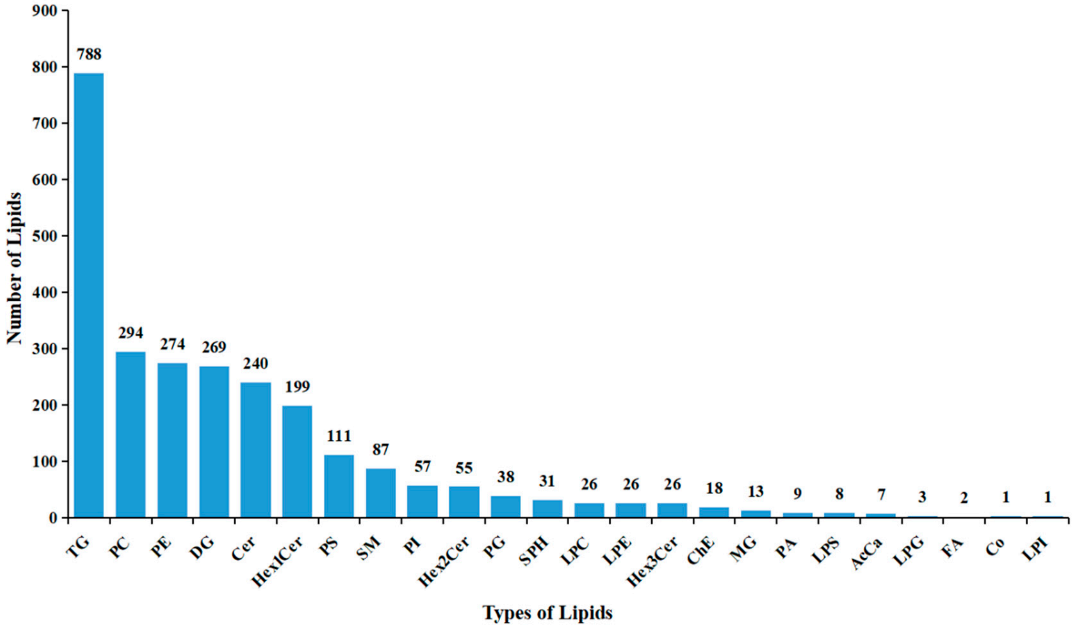

Figure S1. Number of Lipid Molecules

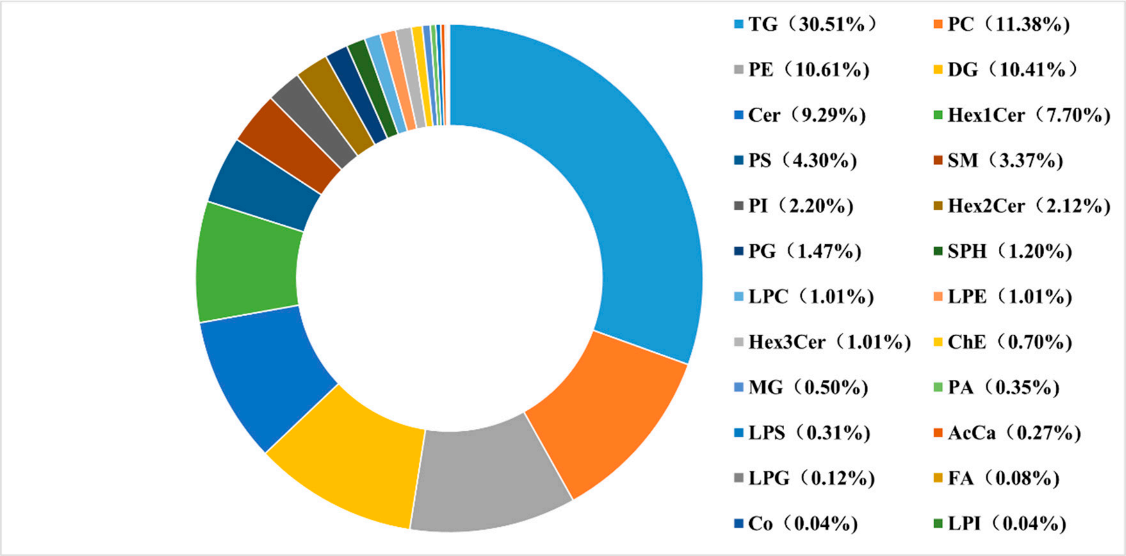

Figure S2. Pie chart of the relative contents of lipid molecules.

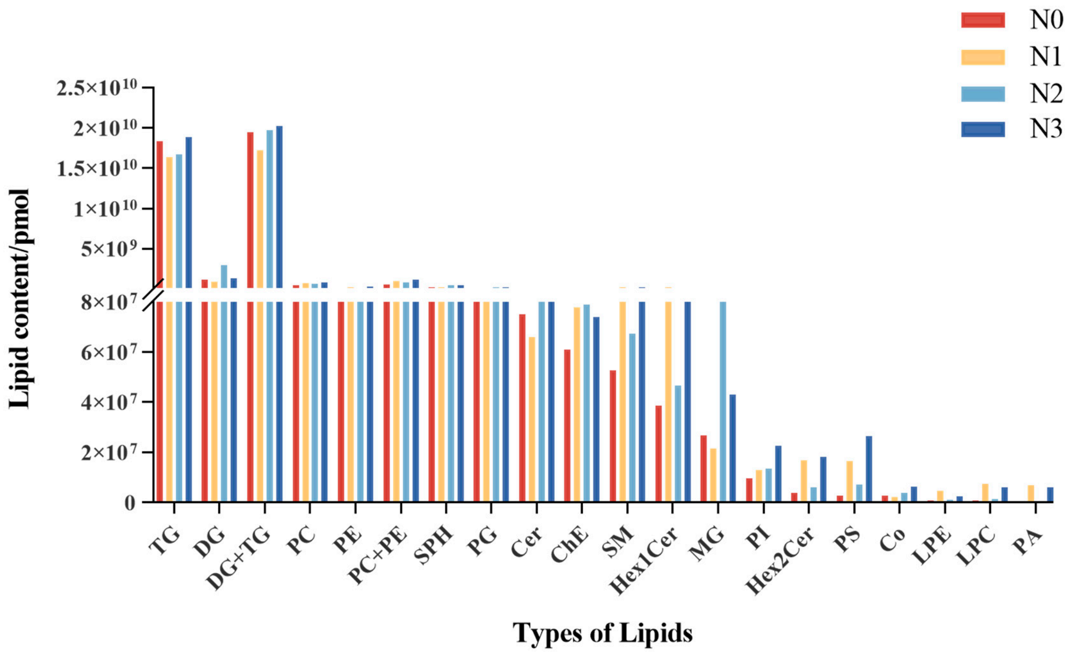

Figure S3. Bar chart of the absolute contents of lipid molecules.

Table S1. internal Standards (SPLASH® LIPIDOMIX MASS SPRCSTANDARD, AVANTI,330707-1EA）

Catalog Number	Mixture Components	Target Concentration (μg/mL)
791637	15:0-18:1(d7) PC	160
791638	15:0-18:1(d7) PE	5
791639	15:0-18:1(d7) PS	5
791640	15:0-18:1(d7) PG	30
791641	15:0-18:1(d7) PI	10
791642	15:0-18:1-d7-PA	7
791643	18:1(d7) LPC	25
791644	18:1(d7) LPE	5
791645	18:1-d7-cholesterol	350
791646	18:1(d7) MG	2
791647	15:0-18:1(d7) DG	10
791648	15:0-18:1(d7)-15:0 TG	55

791649	18:1(d9) SM	30
700041	Cholesterol(d7)	100
